# The LPC-ATX-LPA-LPAR Axis in Major Depressive Disorder: From PC/LPC Metabolism to Receptor-Active Lipid Signaling

**DOI:** 10.3390/ijms27135981

**Published:** 2026-07-03

**Authors:** Weili Wei, Rui Liu, Dan Su, Yuhui Ping, Yonggui Song, Zhifu Ai

**Affiliations:** College of Traditional Chinese Medicine, Jiangxi University of Chinese Medicine, Nanchang 330004, China; vivily24@163.com (W.W.);

**Keywords:** depression, glycerophospholipid metabolism, lysophosphatidylcholine, autotaxin, lysophosphatidic acid, LPA receptor, blood–brain barrier (BBB), MFSD2A, synaptic excitability

## Abstract

Major depressive disorder (MDD) is not reducible to a single neurotransmitter deficit. Current explanations commonly involve monoaminergic dysfunction, hypothalamic–pituitary–adrenal axis dysregulation, immune-inflammatory activation, impaired neuroplasticity and synaptic dysfunction, together with metabolic and neurovascular abnormalities. Lipidomic studies have repeatedly identified glycerophospholipid abnormalities in MDD, but their mechanistic meaning remains unresolved because changes in bulk lipid abundance do not explain how altered lipid metabolism becomes a receptor-level neural signal. This review develops a testable interpretation of the lysophosphatidylcholine (LPC)–autotaxin (ATX)–lysophosphatidic acid (LPA)–LPA receptor (LPAR) axis in which LPC species generated during phospholipid turnover provide ATX substrates, ATX activity determines local LPA generation, LPA production and inactivation shape ligand availability, and LPAR signaling links the lipid product to neural output. This structure shifts the focus from total lipid abundance to matched assessment of lipid species, enzyme activity, anatomical site and receptor subtype. Human studies report lower serum and cerebrospinal fluid (CSF) ATX in MDD, lower CSF LPA 22:6 in MDD and schizophrenia, and negative total LPA findings that caution against biomarker oversimplification. Depression-relevant and broader stress- or anxiety-related experimental studies show that ATX, LPA and LPAR perturbation can affect hippocampal function, synaptic physiology, emotional behavior and stress resilience. The key unresolved issue is whether brain-accessible LPC species, active ATX, locally generated LPA, LPA inactivation capacity and receptor-specific output can be demonstrated within the same MDD-relevant fluid, brain-interface site or neural circuit. Future work should therefore move from fluid-level association toward pathway closure through targeted and spatial lipidomics, anatomical ATX activity mapping, LPA inactivation assays, blood–brain barrier (BBB)/interface analysis, LPAR perturbation and matched circuit or behavioral readouts.

## 1. Introduction

Major depressive disorder (MDD) cannot be adequately explained by monoamine deficiency alone. Monoaminergic mechanisms remain clinically relevant, but they do not account for the delayed onset of many antidepressant responses, treatment resistance, hypothalamic–pituitary–adrenal axis dysregulation, inflammatory comorbidity, metabolic dysregulation, neurovascular abnormalities or impaired synaptic plasticity that are repeatedly described in contemporary depression research [1]. The biological problem is therefore broader than transmitter depletion. MDD is better approached as a disorder in which several regulatory systems converge on altered brain function.

Lipid metabolism has become one of the most informative entry points into this broader view. Reviews of depression lipid biology have described abnormalities across several lipid classes and processes, including glycerophospholipids, sphingolipids, fatty-acid-related metabolites, altered membrane lipid composition, phospholipid turnover and lipid-associated signaling [2,3]. Clinical lipidomic studies have separately reported MDD-associated abnormalities in oxidized fatty acids and acylcarnitines [4], as well as broader serum lipidome changes in real-world patient cohorts [5]. These observations matter because lipids contribute to membrane architecture, inflammatory mediator generation, lipoprotein transport, neurovascular exposure and receptor signaling [2,3]. However, an MDD-associated lipidomic finding does not by itself establish a disease mechanism. A change in lipid abundance may reflect altered synthesis, degradation, transport, carrier distribution or tissue turnover, but it does not necessarily explain how a cellular response is produced.

Phosphatidylcholine (PC) and related membrane phospholipids can generate lysophospholipid intermediates through phospholipase-dependent hydrolysis, including phospholipase A (PLA)-related reactions [6,7]. Oxidative modification can also contribute to lysophospholipid formation under oxidative conditions [7]. Such intermediates do not have the same relationship to downstream lipid signaling. Some primarily reflect membrane phospholipid turnover or oxidative lipid modification, whereas others can serve as mobile substrates for extracellular enzymatic LPA generation. This distinction creates a useful question for depression lipidomics: which lipid changes mainly describe altered membrane phospholipid turnover or oxidative modification, and which changes can be linked to enzymatic LPA generation, receptor activation, and functional neural effects.

Lysophosphatidylcholine (LPC) is central to that question. Psychiatric fluid studies have linked cerebrospinal fluid (CSF) LPA 22:6 to CSF LPC 22:6 [8], reported a positive correlation between serum total LPA and serum total LPC within the MDD group despite no significant group difference [9], and identified an LPA 16:1-containing multilipid serum signature in drug-free female MDD cohorts [10]. These studies position LPC-related metabolism as a relevant entry point for mechanistic analysis because they point to specific LPC-LPA relationships rather than only broad lipid-class changes. LPC also occupies a specific biochemical position. It can arise from PC turnover and PLA-related phospholipid hydrolysis [6,7], and it can then serve as the substrate for a subsequent enzymatic step that generates LPA [11]. Its brain-interface relevance depends on molecular species and transport compatibility rather than on total LPC concentration alone [12].

**Figure 1:**
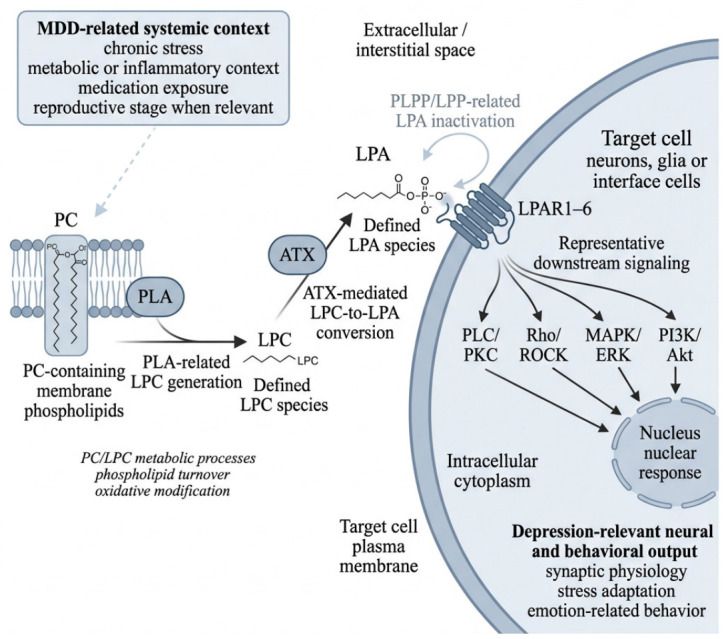
A schematic overview of the PC-PLA-LPC-ATX-LPA-LPAR signaling system. The figure illustrates PLA-related LPC generation, ATX-mediated LPA production, local LPA inactivation and LPAR1 to LPAR6 receptor signaling in relevant cell types. Through these receptors, LPA may engage downstream signaling pathways related to synaptic physiology, stress adaptation and emotion-related behavior.

This subsequent enzymatic step is mediated by autotaxin (ATX, also known as ENPP2), which converts LPC into lysophosphatidic acid (LPA), a receptor-active lysophospholipid [11]. LPA acts through G-protein-coupled LPA receptors and can engage calcium mobilization, PLC/PKC, MAPK, PI3K/Akt and Rho-family signaling [13,14]. These signaling programs are not depression-specific by themselves, but they can influence synaptic organization, neural excitability and stress-related adaptation. The LPC-ATX-LPA-LPAR axis therefore connects membrane phospholipid turnover, PLA-related LPC generation, LPC substrate availability, ATX-mediated LPA generation and LPAR-mediated neural effects. A schematic overview of this sequence is shown in Figure 1.

The present review uses this sequence to distinguish descriptive lipid abnormalities from testable lipid-signaling mechanisms. In this view, LPC-related lipid alterations become more informative when defined LPC species can be linked to ATX activity, specific LPA molecular species, local LPA production and inactivation, LPAR subtype signaling and matched neural or behavioral output within the same biological context. Previous reviews have summarized LPA receptor biology in mood regulation and the broader involvement of LPA signaling in neuropsychiatric and neurodegenerative disorders [15,16]. Building on these reviews, the present review moves upstream from receptor-centered LPA signaling to depression-associated PC/LPC metabolic changes, LPC availability, ATX-mediated LPA production, LPA inactivation and LPAR-related neural output in MDD-relevant settings.

## 2. Review Scope and Interpretive Boundaries

This article is a hypothesis-generating critical narrative synthesis. It uses purposive literature sampling rather than a PRISMA-based systematic review or meta-analysis. The objective is not to calculate pooled effect sizes or rank therapeutic interventions. Instead, the objective is to organize an emerging literature around a testable mechanistic question and to define the evidence required before the LPC-ATX-LPA-LPAR sequence can be treated as a pathway-level mechanism in depression.

The literature was identified from PubMed, Web of Science, Scopus and Google Scholar from database inception to 12 May 2026. Search terms included depression, major depressive disorder, anxiety, chronic stress, glycerophospholipid, lysophosphatidylcholine, LPC, autotaxin (ATX, ENPP2), lysophosphatidic acid, LPA, LPA receptor, LPAR1, LPAR2, LPAR3, LPAR5, PRG-1, major facilitator superfamily domain-containing 2A (MFSD2A), blood–brain barrier, phospholipase A2, lipidomics, perimenopause, postpartum depression, estrogen, progesterone, neurosteroid, antidepressant and Chinese herbal formula. Reference lists from key mechanistic studies and recent reviews were also considered.

Studies were evaluated according to diagnostic relevance, sample type, molecular specificity and mechanistic proximity to the proposed sequence. Human studies in clinically diagnosed MDD cohorts, or psychiatric fluid studies that included MDD patients, were treated as direct clinical evidence. CUMS and other depression-like behavioral models were treated as depression-relevant experimental evidence because they provide mechanistic information on ATX/LPA/LPAR signaling, synaptic plasticity and behavioral readouts, but they do not replace clinical MDD data. Studies focused on anxiety-like behavior, stress responses, receptor-deficiency phenotypes, synaptic physiology or stress resilience were interpreted as broader mood- or stress-related evidence. Endocrine-transition and pharmacological studies were considered contextual or interventional evidence only when they helped clarify how the pathway could be tested.

## 3. From Depression-Related PC/LPC Metabolism to the LPC-ATX-LPA-LPAR Hypothesis

### 3.1. Why LPC-Related Metabolism Narrows the Field Toward the LPC-ATX-LPA-LPAR Axis

Depression-associated lipid changes are heterogeneous, and that heterogeneity is biologically meaningful. Reviews of depression lipid biology show that reported lipid findings span multiple classes, including glycerophospholipids, sphingolipids, fatty-acid-related metabolites and other lipid-signaling systems [2,3]. Case–control lipidomics has also identified distinct abnormalities in oxidized fatty acids, acylcarnitines and broader serum lipid profiles [4,5]. Such findings are valuable for phenotype mapping, but they do not automatically identify a mechanistic axis. A review that seeks a pathway-level explanation must therefore prioritize lipid changes that not only recur in depression-relevant studies but also connect to a chemically defined downstream signaling route.

Glycerophospholipid and lysophospholipid metabolism meet the first condition and create a rational search space for the second. PC and related phospholipids are major membrane constituents, and PC turnover or PLA-related hydrolysis can generate lysophospholipids with distinct acyl-chain compositions and biological behavior [6,7]. This is important because a bulk decline or increase in a phospholipid class can conceal opposite changes among individual molecular species. The same caution applies to LPC. Total LPC is an analytically convenient measure, but it is a poor substitute for the availability of specific LPC species that can enter transport routes, encounter ATX or participate in receptor-active LPA generation.

LPC is relevant to this review because it is the substrate from which ATX generates LPA. This biochemical relationship allows depression-related LPC findings to be evaluated in relation to defined LPA species and LPA receptor-mediated outcomes. Omori et al. reported lower CSF LPA 22:6 in MDD and schizophrenia, and this signal related to LPC 22:6 rather than to total LPA measures [8]. Riya et al. found no group-level difference in serum total LPA or LPC between MDD patients and controls, but they observed a positive correlation between the two lipid classes within the MDD group [9]. Kim et al. identified LPA 16:1 within multilipid panels that differentiated current MDD, remitted MDD and controls in drug-free female cohorts [10]. These studies do not establish a disease pathway, but they collectively show why the field must move beyond total lipid abundance and toward molecular species with interpretable biochemical relationships.

LPC is also positioned at a transport interface. Nguyen et al. showed that MFSD2A transports DHA in LPC-linked form and also transports selected long-chain plasma LPC species, including LPC-oleate and LPC-palmitate, with lower transport capacity than LPC-DHA [12]. In the same study, LPC species with acyl chains shorter than C14 were not efficiently transported by MFSD2A [12]. Structural work later clarified how substrate binding can drive conformational transitions in this transporter [17]. Independently, Mfsd2a loss disrupts blood–brain barrier (BBB) formation and increases endothelial transcytosis, showing that its transport function sits within a broader barrier-regulatory program [18]. These findings do not show that depression-associated LPC abnormalities automatically enter the brain. They show something more important for this review: peripheral “LPC” is not a uniform CNS input. The probability that a circulating LPC species alters brain substrate availability depends in part on molecular species and transport compatibility. This point directly strengthens the case for species-resolved rather than total-LPC interpretation.

### 3.2. Autotaxin-Mediated Generation of LPA from LPC

ATX is important because it catalyzes the conversion of LPC to LPA, a lipid mediator that can activate LPA receptors. Umezu-Goto et al. established that ATX has lysophospholipase D activity and generates LPA from LPC [11]. This reaction links changes in specific LPC species to the production of LPA, a receptor-active lipid mediator. Once LPC is considered as an ATX substrate, the relevant question is not only whether LPC is increased or decreased, but also whether a specific LPC species is present in the same tissue or cellular setting as catalytically active ATX.

ATX biology also argues against interpreting circulating ATX levels as a direct substitute for local ATX-mediated LPA production or receptor signaling. Fulkerson et al. showed that ATX can bind beta1 and beta3 integrins, thereby localizing LPA production to cell surfaces [19]. Salgado-Polo et al. later demonstrated that ATX facilitates selective LPA receptor signaling [20]. For depression research, this means that serum ATX, CSF ATX, brain-region ATX expression and spatial ATX activity are related readouts, but they are not interchangeable.

This distinction helps explain why human and experimental findings may appear inconsistent. A patient can have reduced CSF ATX abundance without a parallel measurement of parenchymal ATX activity. Reduced CSF ATX levels in a human study do not necessarily indicate reduced ATX activity in brain parenchyma. A mouse brain homogenate can show lower hippocampal ATX without revealing whether a microdomain near synapses or a vascular interface is locally enriched or depleted. Similarly, lower hippocampal ATX measured in a brain homogenate does not identify the cell type or anatomical interface in which ATX activity is altered. Serum and CSF measurements are clinically informative, but they cannot alone define the anatomical site or functional consequence of LPC-to-LPA conversion.

### 3.3. LPA Receptors and Mood-Relevant Neural Signaling

The LPC-ATX-LPA-LPAR framework becomes neurobiologically relevant only when LPA production is linked to receptor-mediated output. Six major LPA receptors, LPAR1 through LPAR6, couple to overlapping but non-identical G-protein programs and thereby influence calcium mobilization, PLC/PKC activity, MAPK signaling, PI3K/Akt signaling and cytoskeletal regulation [13,14]. A receptor-based account must therefore specify more than “LPA changes.” It must ask which LPA species changes, which receptor subtype is available in the relevant cell population and which functional output follows.

Among receptor subtypes, LPAR1 currently has the broadest mood-related evidence base. Consistent with the receptor-focused mood literature, a recent systematic review identified LPAR1 as the most recurrent receptor subtype linking LPA biology to emotional regulation [15]. Primary studies then show that LPA1 deficiency or blockade can modify hippocampal LPA species [21], alter stress-related behavior and ventral hippocampal excitatory–inhibitory gene profiles [22], and induce depression-like behavior with related changes in brain functional activity [23]. Other models connect LPA/LPA1 perturbation to adult hippocampal neurogenesis [24], GABAergic deficits and coping abnormalities [25], altered HPA-axis regulation [26], and sex-dependent emotional phenotypes [27]. However, mood-relevant LPA biology is not limited to LPAR1. PRG-1, also known as PLPPR4, is a brain-enriched phospholipid phosphatase-related membrane protein that regulates lipid-phosphate-mediated signaling at glutamatergic synapses. Trimbuch et al. showed that PRG-1 restrains extracellular lipid-phosphate signaling at glutamatergic synapses and that loss of PRG-1 increases excitatory transmission through a presynaptic LPA2-dependent mechanism [28]. Tüscher et al. further linked PRG-1 dysfunction to intermediate psychiatric phenotypes in human carriers and stress-related phenotypes in mice; selected mouse phenotypes were normalized by ATX inhibition [29]. Together, these findings show that local lipid-phosphate-mediated signaling can influence excitatory synaptic output and psychiatric-relevant phenotypes through mechanisms that are not limited to LPAR1.

Neuronal physiology studies provide functional evidence that LPA can regulate hippocampal synaptic activity. Brandt et al. reported that LPA selectively modulates excitatory transmission and intracellular calcium responses in hippocampal neurons [30]. This study does not show that hippocampal LPA signaling is pathogenic in MDD. It shows that LPA can influence hippocampal synaptic output. Depression-related LPC, ATX and LPA findings should therefore be interpreted by testing whether molecular changes are linked to receptor-specific and circuit-relevant outcomes, rather than by expecting identical directional changes across serum, CSF and brain tissue.

## 4. Current Evidence Across Human and Experimental Studies

### 4.1. MDD and Psychiatric Fluid Evidence

Human studies are clinically relevant, but serum and CSF measurements usually cannot identify the tissue source of ATX or the anatomical site of LPA receptor activation. Itagaki et al. reported lower serum ATX in 37 patients with MDD undergoing electroconvulsive therapy than in 47 nondepressed controls. In a separate sample, CSF ATX was also lower in 26 MDD patients than in 27 controls [31]. Serum ATX increased after electroconvulsive therapy and was inversely related to depressive symptom burden before treatment [31]. These findings support a state-linked alteration in measured ATX levels in MDD.

Omori et al. provided molecular-species-level evidence by measuring CSF LPA 22:6 rather than relying only on total LPA. They found lower CSF LPA 22:6 in patients with MDD and schizophrenia than in healthy controls, whereas total CSF LPA was less informative [8]. The same study reported that CSF LPA 22:6 was associated with LPC 22:6. However, CSF ATX activity and phospholipid phosphatase 1 (PLPP1) did not differ significantly across groups, and LPA 22:6 did not correlate with ATX activity in the patient groups [8]. Together, these results indicate that a specific CSF LPA species can be altered without supporting a simple model in which lower bulk CSF ATX activity directly explains lower CSF LPA 22:6.

The same findings also expose a second interpretive gap: local LPA signaling is determined by both production and extracellular inactivation. Direct in vivo evidence shows that LPP1/PLPP1 degrades extracellular LPA and contributes to its clearance [32]. Omori et al. measured CSF PLPP1 and found no significant between-group difference among MDD, schizophrenia and control cohorts [8]. This negative result does not support a simple CSF-level PLPP1 explanation for reduced LPA 22:6. Whether PLPP1 or related PLPP enzymes contribute to local LPA signaling abnormalities within mood-relevant CNS regions remains unresolved.

Negative and mixed biomarker studies provide equally important constraints. Gotoh et al. found no association between total LPA in CSF or plasma and MDD diagnosis, symptom severity or psychotropic medication [33]. Riya et al. reported no significant between-group difference in serum total LPA or LPC, even though LPA and LPC correlated positively within the MDD group [9]. These findings should not be dismissed as uninformative. They argue against using total circulating LPA or LPC as general diagnostic surrogates for depression-relevant receptor signaling.

Kim et al. offer a complementary perspective. In female drug-free subjects, LPA 16:1 contributed to multilipid panels that distinguished current MDD, remitted MDD and controls [10]. It nevertheless supports the need for molecular-species-level lipid analysis, because depression-relevant information may be lost when measurements are collapsed into total LPA or total LPC. This finding supports the value of molecular-species-level lipid analysis in MDD, but as part of a multilipid panel rather than as a stand-alone biomarker.

### 4.2. Depression-Relevant Experimental Models and Broader Stress- or Anxiety-Related Evidence

Experimental models provide functional information that human fluid studies cannot provide, but they differ in their relevance to MDD. Chronic unpredictable mild stress (CUMS) and related depression-like behavioral paradigms are treated here as depression-relevant models because they assess stress-induced anhedonia, behavioral despair or related affective changes. Broader stress, anxiety and receptor-deficiency models are interpreted separately because they mainly inform receptor function, stress vulnerability or circuit regulation rather than evidence from MDD patient cohorts. In a CUMS model, Wang et al. reported reduced hippocampal ATX and LPA. Hippocampal adeno-associated virus-mediated ATX (AAV-ATX) supplementation improved depression-like behavior and restored synaptic-plasticity-related molecular readouts [34]. This study links a regional ATX/LPA deficit to behavioral and molecular outcomes in a depression-relevant model. The upstream LPC source was not tested.

Receptor-focused studies provide broader mood- and stress-related evidence rather than direct MDD evidence. Tabbai et al. demonstrated that acute stress and LPA1 deficiency reshape hippocampal LPA species, with anxiety-related behavior associated with LPA 16:0 and locomotor measures associated with LPA 18:0 [21]. Moreno-Fernández et al. reported that the LPA-LPA1 pathway modifies stress-related behavior and ventral hippocampal excitatory–inhibitory gene profiles [22]. In a separate study, genetic deletion or acute pharmacological blockade of LPA1 induced depression-like behavior with related changes in brain functional activity [23]. Together, these studies support a role for LPA1-related signaling in stress- and mood-relevant neural regulation.

Longer-term ligand and receptor manipulations reinforce this conclusion. Chronic central C18:1 LPA administration produced antidepressant-like effects and enhanced adult hippocampal neurogenesis, whereas chronic antagonism of LPA1-3 receptors induced anxiety- and depression-like behaviors and reduced neurogenesis [24]. In a related line of work, LPA1 deficiency was linked to GABAergic deficits, anxiety-like behavior and coping abnormalities, and interneuron precursor transplantation partially improved the phenotype [25]. These data indicate that LPA signaling contributes to circuit balance and adaptive behavior, but that its direction of effect depends on receptor subtype, ligand exposure pattern and neural substrate.

Recent studies add endocrine, sex-stratified and translational dimensions. Moreno-Fernández et al. linked LPA1 deficiency to social avoidance, impaired dexamethasone suppression and abnormal corticosterone rhythmicity [26]. Sánchez-Marín et al. reported sex-dependent emotional phenotypes and region-dependent neurotransmitter-related transcriptional changes in LPA1-deficient mice [27]. Larrieu et al. found higher serum LPA 16:0 in high-risk susceptible humans and high-anxiety mice, and they showed that platelet-derived LPA 16:0 reduces adult hippocampal neurogenesis and stress resilience through LPA1-dependent mechanisms [35]. These findings indicate that endogenous LPA species can be associated with trait-anxiety and stress-resilience-related phenotypes.

Taken together, the evidence reviewed in Section 4.1 and Section 4.2 spans human fluid studies, depression-relevant experimental models and broader receptor-focused systems. Section 4.3 therefore addresses why these findings should be interpreted according to measured readout, sample type, anatomical site and receptor context. This evidence architecture is summarized in Table 1.

### 4.3. Sources of Divergent Findings: Molecular Species, Sample Type, Anatomical Site and Receptor Context

Findings in this area should first be separated according to the measured variable and the sample in which it was measured. In human MDD cohorts, reported readouts include serum or CSF ATX abundance [31], CSF LPA 22:6 [8], total LPA in CSF or plasma [33], serum total LPA and total LPC [9], and serum lipidomic signatures containing LPA 16:1 in drug-free female MDD cohorts [10]. These readouts should not be treated as interchangeable. Total LPA does not indicate whether a defined LPA species is altered. Total LPC does not identify which LPC species may be available for ATX-mediated conversion. ATX abundance is also distinct from ATX enzymatic activity.

These findings should be interpreted according to what was measured and where it was measured. A negative association between total LPA in plasma or CSF and MDD diagnosis does not directly test whether a specific LPA species, a brain region or a receptor-expressing cell population is altered. Conversely, a species-specific lipid change or receptor-level experimental result should not be used to claim a completed LPC-ATX-LPA-LPAR pathway unless relevant LPC species, ATX-mediated LPA production, LPA inactivation and receptor-mediated output are assessed in the same biological setting.

**Figure 2:**
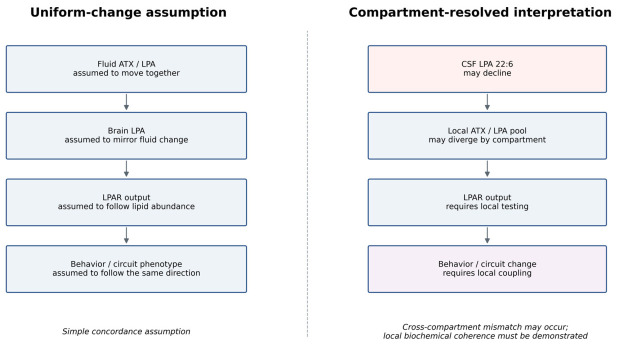
Uniform-change assumption versus compartment-resolved interpretation. A single-direction model expects fluid ATX/LPA changes, brain LPA changes, receptor output and behavioral outcomes to move together. The evidence reviewed here does not support that assumption. A compartment-resolved interpretation allows cross-compartment mismatch but requires local biochemical coherence: a defined LPC/LPA species, catalytically active ATX and receptor-specific output must be demonstrated within the relevant CNS context.

Experimental and translational studies therefore provide complementary functional evidence, but they do not directly explain serum or CSF findings in MDD patients. Hippocampal ATX/LPA rescue in CUMS mice supports the relevance of regional ATX/LPA signaling to depression-like behavior [34]. LPA1-focused studies then demonstrate receptor-dependent effects on stress behavior and excitatory–inhibitory gene programs [21,22], depression-like behavior with altered functional brain activity [23], adult neurogenesis [24], inhibitory-circuit integrity [25], endocrine stress regulation [26], and sex-dependent emotional phenotypes [27]. The anxiety-linked LPA 16:0 findings further show that an endogenous circulating LPA species can be associated with trait-anxiety or stress-resilience-related phenotypes [35]. Together, the current evidence supports the relevance of specific lipid species, regional ATX/LPA changes and receptor-linked behavioral effects, but it does not yet establish a continuous LPC-ATX-LPA-LPAR pathway connecting MDD patient fluids, brain tissue and behavior. This interpretation is summarized in Figure 2, which contrasts a uniform-change assumption with a sample- and site-resolved interpretation.

## 5. The Decisive Missing Link: Spatial Convergence of Brain-Accessible LPC Species and Catalytically Active ATX

The central evidence gap is now sharply defined. The unresolved problem is not whether ATX can generate LPA. That point is established biochemically [11]. The unresolved problem is not whether LPA signaling can alter synaptic physiology. PRG-1/LPA2 work demonstrates lipid-phosphate control of excitatory transmission [28,29], and direct neuronal experiments show that LPA modulates hippocampal excitatory transmission and intracellular calcium responses [30]. The unresolved problem is also not whether LPA receptor perturbation can influence emotion-related or stress-related phenotypes. LPA1-focused studies have shown effects on hippocampal LPA species [21], chronic-stress behavioral and transcriptional responses [22], depression-like functional brain activity [23], neurogenesis [24], GABAergic circuitry [25], HPA-axis regulation [26], sex-dependent emotional phenotypes [27], and anxiety-related vulnerability [35]. The unresolved question is whether, within a predefined MDD-relevant sample type, CNS interface or mood-related brain region, a defined LPC species, catalytically active ATX, locally generated LPA, local LPA inactivation capacity and receptor-specific neural output can be demonstrated in a temporally or experimentally ordered design.

Human fluid studies reveal why this gap matters. Lower serum and CSF ATX in MDD indicate altered ATX biology in psychiatric illness [31]. Lower CSF LPA 22:6 indicates a species-specific lysophospholipid abnormality [8]. Yet neither finding localizes the ATX source, identifies the LPC source or proves where receptor-active LPA is generated. Omori et al. further showed that CSF LPA 22:6 did not correlate with measured CSF ATX activity in the patient groups [8]. This result prevents a direct leap from fluid abnormality to local catalytic mechanism.

Central ATX anatomy is more complex than a single “brain ATX” variable suggests. Savaskan et al. reported ATX expression in leptomeningeal cells and oligodendrocyte precursor cells, with reactive astrocytic upregulation after neurotrauma [36]. Tachikawa et al. later found high ATX expression in the choroid plexus of developing and adult mouse brain, with predominant localization at the cerebrospinal-fluid-facing apical side of choroid-plexus epithelial cells [37]. These data imply that CSF-facing ATX, meningeal ATX, glial ATX and parenchymal ATX should not be treated as interchangeable pools. They may differ in substrate access, local LPA generation and relevance to neural circuits.

The LPC side of the pathway is equally unresolved. MFSD2A-mediated transport establishes that CNS substrate access is selective rather than generic. The strongest evidence concerns LPC-DHA, although selected long-chain LPC species such as LPC-oleate and LPC-palmitate can also be transported with lower capacity [12]. Stress-related BBB dysfunction and higher regional BBB permeability in patients with MDD establish a separate interface abnormality that may alter exposure to circulating molecules [38,39]. Local PC turnover or PLA-related hydrolysis within neural and glial membranes provides yet another potential LPC source [6,7]. These routes are mechanistically distinct. Regulated transport, barrier leakage and local membrane phospholipid turnover should not be compressed into one generic statement that peripheral LPC reaches the brain.

This anatomical uncertainty explains why future studies must combine source mapping with functional testing. CSF can provide an interface-proximal readout, but it cannot substitute for brain-region-specific receptor output. Brain homogenates can show that an analyte is present, but they can erase perivascular, choroid-plexus, synaptic or glial microenvironments. The key experiment is not simply to measure more ATX or more LPA. It is to determine how the local LPA pool is maintained in a defined CNS interface or mood-related brain region. For an ATX-centered LPC-to-LPA mechanism, the relevant LPC species and catalytically active ATX should be spatially linked because this is the condition under which local ATX-mediated conversion can be inferred. However, local LPA availability should not be attributed to ATX activity alone. It may also be influenced by LPA inflow or outflow from adjacent fluid or vascular-interface spaces, carrier-bound LPA in biological fluids, local LPA degradation, and upstream PLA1/PLA2-dependent changes in the LPC substrate pool.

The pathway becomes stronger only when the following sequence is demonstrated within one model or cohort: a defined LPC species is available at a relevant CNS interface or parenchymal compartment; catalytically active ATX is spatially linked to that LPC species when local ATX-mediated conversion is proposed; a defined LPA species is generated; local LPA degradation and possible LPA inflow or outflow are considered; PLA1/PLA2-related upstream generation of the LPC substrate pool is assessed when this upstream LPC-generating process is part of the model; a defined LPAR subtype and downstream signaling program respond; and the resulting output tracks with circuit or behavioral change. Without that sequence, the literature supports an important framework, but not a completed CNS production route. These requirements are summarized in Table 2.

## 6. Pharmacological Relevance: Intervention Clues Without Premature Mechanistic Closure

Pharmacological evidence strengthens the biological interest of the pathway, but it should be used with restraint. Kajitani et al. reported that amitriptyline directly binds LPAR1 and acts as a G protein-biased LPAR1 agonist [40]. The same study found that this biased agonism characterized tricyclic antidepressants but not SSRIs, SNRIs, ketamine, vortioxetine or trazodone [40]. This finding matters because it establishes a direct receptor-level connection between an established antidepressant class and LPA signaling. It does not show that ATX activity or circulating LPC/LPA species predict treatment response, and it does not prove that tricyclic efficacy in patients is mediated primarily through LPAR1.

Multi-component interventions provide a different kind of clue. Chaihu-Shu-Gan-San altered phospholipid and bile-acid metabolism in a chronic unpredictable stress rat model [41]. Xiao-Yao-San reduced stress-associated BBB injury through glucocorticoid receptor-mediated upregulation of occludin in experimental systems [42]. Saikosaponin-d was linked to LPA1-associated neuronal apoptotic signaling in vitro [43]. A perimenopausal depression model further used a brain–liver communication framework to examine Chaihu-Shugan-San [44]. These studies do not close the LPC-ATX-LPA-LPAR chain. They indicate that metabolism, barrier integrity and LPA-related receptor biology are experimentally modifiable in settings relevant to stress and affective vulnerability.

Yueju Pill provides a particularly useful example of how a formula-level intervention could be repurposed as a mechanistic tool. Prior work supports rapid antidepressant-like effects and acute enhancement of brain BDNF expression after Yueju Pill administration [45]. Those findings do not establish ATX-LPA involvement. However, if a future study were to pair Yueju Pill or one of its representative constituents with targeted PC/LPC/LPA lipidomics, spatial ATX activity mapping and LPAR perturbation, it could test whether a multi-component intervention acts upstream, within or outside the proposed axis. The intervention becomes informative only when it is embedded in a causal design rather than treated as indirect confirmation by behavioral improvement alone.

## 7. Criteria for Establishing, Constraining or Refuting the Pathway

The field now needs integrated pathway-level testing rather than additional disconnected associations. A persuasive animal experiment should combine a disease-relevant condition, such as chronic stress, with reproductive hormone-transition paradigms added only when the study question requires them. Within the same model, several linked layers should be measured: targeted PC, LPC and LPA molecular species; BBB or vascular-interface status; ATX localization and enzymatic activity; local LPA inactivation capacity, where relevant; LPAR subtype expression and downstream signaling; and circuit-level or behavioral readouts. The same model should then include intervention experiments rather than relying only on correlations. PLA1 or PLA2 abundance and activity could be assessed to determine whether upstream PLA-related LPC generation changes the LPC substrate pool available for ATX-mediated LPA generation. ATX inhibition, ATX gain-of-function or labeled LPC tracing could then test whether LPC-to-LPA conversion occurs in the implicated tissue site or vascular-interface region. PLPP1 or related LPA-dephosphorylating pathways should be examined when altered LPA clearance is suspected. When local LPA levels are inferred from fluid samples or vascular-interface measurements, LPA delivery, removal and carrier-associated transport should also be considered. LPAR subtype blockade or genetic disruption, combined with electrophysiology or calcium imaging, could test whether the relevant receptor subtype is required for cellular output, circuit function or behavioral change.

Human studies require a parallel but ethically adapted structure. A strong MDD cohort would include diagnostic characterization, symptom measures, antidepressant exposure or other psychotropic medication status, metabolic status, targeted LPC and LPA species, inflammatory markers and, where feasible, neurovascular or BBB-related imaging. In cohorts including women across reproductive or menopausal stages, reproductive stage and sex-steroid status should be recorded as participant characteristics or covariates, not treated as direct evidence for the axis. CSF LPA species can be informative because MDD-associated LPA 22:6 differences have been detected in this compartment [8]. CSF ATX abundance may also be relevant because reduced CSF ATX has been reported in MDD [31]. Yet recent anatomical work on choroid-plexus ATX indicates that CSF-facing ATX should be interpreted as an interface-proximal signal rather than as a direct surrogate for hippocampal or prefrontal receptor activity [37]. The purpose is not to force all compartments into one direction of change. The purpose is to determine whether a coherent substrate-enzyme-product-inactivation-output relationship emerges within a biologically defined state.

**Figure 3:**
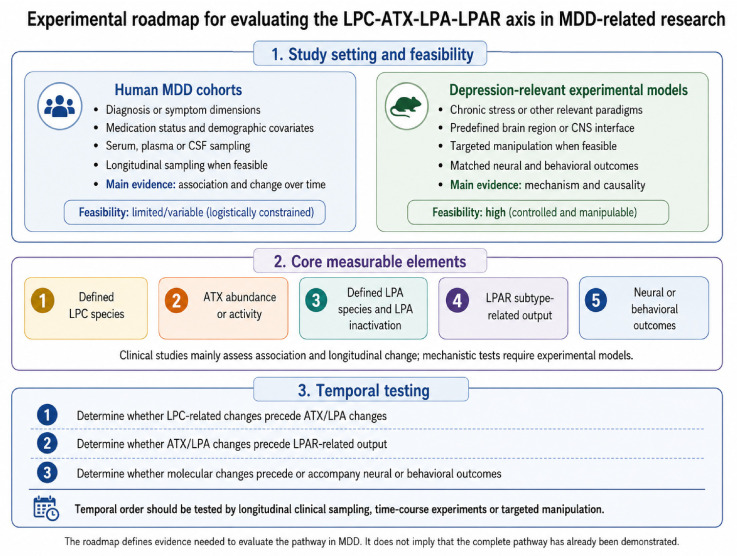
An experimental roadmap for evaluating the LPC-ATX-LPA-LPAR axis in MDD-related research. The roadmap distinguishes clinically feasible measurements in human MDD cohorts from mechanistic tests that require depression-relevant experimental models. It also indicates that temporal order should be examined by longitudinal clinical sampling, time-course experiments or targeted manipulation. The figure does not imply that the complete pathway has already been demonstrated in MDD.

The pathway should also be falsifiable. If a predicted endocrine or stress condition changes mood-relevant behavior without altering targeted LPC/LPA species, interface markers, spatial ATX activity or receptor output in the proposed compartment, the pathway is weak in that model. If a plasma or CSF LPC species relates to brain exposure but not to local LPA generation, ATX activity or receptor signaling, substrate availability alone is insufficient. If ATX inhibition or labeled substrate tracing fails to alter the predicted local LPA species, local ATX-dependent conversion should not be treated as necessary. A framework that cannot fail is not mechanistically useful; this one can fail, and that is precisely why it is worth testing. Figure 3 summarizes this validation logic by separating clinically feasible measurements from mechanistic tests that require depression-relevant experimental models and by indicating where temporal order would need to be examined.

Table 3 is based on a falsifiability logic rather than on pooled statistical estimation. A formulation was considered testable when it specified the biological setting, measured variables, comparison or temporal design, and a result that would weaken the proposed interpretation. Broad statements were considered non-testable when they did not define the measured lipid species, ATX readout, anatomical site, receptor output or expected direction of evidence.

## 8. Discussion

The value of the LPC-ATX-LPA-LPAR framework is not that it converts every lipid abnormality in depression into one disease mechanism. Its value is that it identifies a specific explanatory gap and defines the type of evidence needed to evaluate it. Depression lipidomics has generated an expanding list of altered molecules, and these findings should not be reduced to LPC and LPA alone. However, a list of altered molecules cannot by itself explain how a metabolic disturbance becomes a receptor-level neural signal. Sphingolipids, fatty-acid-related metabolites, additional lysophospholipids and lipid-sensing receptor systems may each contribute to depression-related biology through inflammatory, membrane-related, neurovascular or synaptic mechanisms. The LPC-ATX-LPA-LPAR axis provides a narrower route for the question addressed here because it can be followed from a membrane-derived substrate to an extracellular converting enzyme, a receptor-active lipid product and receptor-specific neural output. LPC is important in this context because it is both a depression-relevant lysophospholipid and a chemically defined precursor of receptor-active LPA. ATX is important because it makes that conversion local and biologically selective. LPARs are important because they translate the lipid product into signaling programs capable of altering synaptic and circuit function.

This perspective differs from previous reviews that focus on LPA receptors in mood regulation or on LPA signaling across neuropsychiatric and neurodegenerative disorders [15,16]. The current review begins upstream, with depression-associated PC/LPC metabolic changes, and asks which route could plausibly connect these changes to receptor-level output. It then follows the evidence downstream and identifies precisely where the chain remains incomplete. This structure prevents two opposite errors. One error is to overstate fluid abnormalities as proof of a CNS signaling mechanism. The other is to dismiss the pathway because total LPA studies or different compartments do not show uniform directional agreement. Both errors arise from asking measurements to answer questions they were not designed to answer.

The apparent inconsistency of the literature is therefore scientifically productive. MDD studies have reported lower serum ATX and lower CSF ATX [31], lower CSF LPA 22:6 [8], no diagnostic association for total LPA in CSF or plasma [33], and no significant group difference in serum total LPA or serum total LPC [9]. A separate serum lipidomic study identified LPA 16:1 within a multilipid signature in drug-free female MDD cohorts [10]. In parallel, anxiety-focused cross-species work reports higher serum LPA 16:0 in vulnerability states [35]. These observations should not be forced into one simple “up” or “down” narrative. They indicate that the relevant biology is likely to be molecular-species specific, anatomically organized, governed by both LPA production and inactivation, and receptor-context dependent. These observations indicate that future studies should specify the lipid species, sample type, anatomical site, ATX activity, LPA inactivation and receptor-related output being tested, rather than treating all ATX or LPA readouts as equivalent.

The menopausal transition and postpartum period are best positioned as contexts for future study design rather than as established mechanisms within the LPC-ATX-LPA-LPAR axis. Both states involve marked changes in ovarian steroids or neurosteroids and are associated with increased depressive symptoms or depressive disorders in a subset of women [46,47,48]. In the menopausal transition, clinical staging criteria and estradiol treatment response support the recording of reproductive stage and sex-steroid status in depression cohorts [46,49,50]. Clinical imaging evidence also suggests that transdermal estradiol can modulate resting-state connectivity in women with perimenopausal depression [51]. Menopause-associated studies and ovariectomized animal models also report changes in circulating lipids or LPC-related metabolic features [52,53,54]. The postpartum literature adds a related example of reproductive-state neurobiology, because pregnancy-associated GABA_A_ receptor plasticity and neurosteroid-based treatments show that neurosteroid-sensitive mechanisms can be clinically relevant to postpartum depression [47,48,55]. At the molecular level, studies in hormone-responsive peripheral or reproductive tissues indicate that steroid state can regulate PLA2 activity [56,57], Enpp2/ATX expression [58] and LPA3 expression [59]. These observations raise a focused question for future work: whether reproductive stage or sex-steroid status modifies LPC species, ATX activity or abundance, LPA species, LPA inactivation and LPAR-related signaling within the same depression-relevant model or cohort.

The pathway also carries a useful translational discipline. Kajitani et al. demonstrate that LPAR1 is not merely a descriptive receptor but a pharmacologically actionable receptor target for prototypic tricyclic antidepressants [40]. Formula-level and natural-product studies show that stress-relevant phospholipid metabolism [41], BBB integrity [42], LPA1-related neuronal signaling [43], brain–liver communication in a perimenopausal depression model [44], and antidepressant-like responses to Yueju Pill [45] can each be experimentally perturbed. Yet the present review does not treat these observations as clinical validation of the pathway. It treats them as reasons to design better mechanistic studies. Translation becomes credible when intervention, molecular species, anatomical conversion, LPA inactivation and functional output are studied together.

A central conclusion is therefore methodological and biological at the same time. Future studies should test whether the LPC-ATX-LPA-LPAR sequence provides a route by which depression-associated PC/LPC metabolic changes are linked to receptor-level neural output in depression-relevant contexts. If the sequence is not supported under spatial and causal testing, the same evidence would indicate that depression-related lysophospholipid changes are better interpreted as parallel metabolic states rather than as drivers of receptor signaling. Either outcome is informative. The interpretation of bulk lipid abnormalities, local receptor phenotypes and endocrine vulnerability will be stronger when they are examined within a shared experimental design.

## 9. Conclusions

The LPC-ATX-LPA-LPAR axis can be used as a hypothesis-generating framework to evaluate whether specific depression-associated glycerophospholipid or lysophospholipid changes are linked to ATX-mediated LPA production and LPAR-mediated neural outcomes. Its value lies in separating four testable elements: defined LPC species, ATX as the extracellular converting enzyme, defined LPA species and LPAR-mediated outcomes related to synaptic physiology, stress adaptation or emotion-related behavior.

Current evidence supports the biological relevance of individual components of this axis, including species-specific LPA changes, ATX alterations and LPA receptor-related neural or behavioral effects. However, current evidence has not shown that these components form a complete LPC-ATX-LPA-LPAR pathway in depression. A stronger test would need to examine a defined LPC species, ATX activity, local LPA production and inactivation, and LPAR subtype involvement within the same depression-relevant model and a predefined mood-related brain region, such as the hippocampus or prefrontal cortex, together with relevant neural and behavioral outcomes. The order of these events also remains unresolved and would require time-course experiments or targeted manipulation of specific steps in the pathway.

Reproductive stage and sex-steroid status may be recorded in future studies when relevant, but current evidence does not establish them as direct drivers of LPC-ATX-LPA-LPAR signaling in depression. Future studies should therefore prioritize species-resolved lipidomics, anatomical mapping of ATX, assessment of BBB-related LPC transport when relevant, LPAR subtype analysis, causal testing and functional outcomes. Such studies would help determine whether the proposed pathway is supported, limited to specific contexts or not supported, and would clarify how LPC and LPA changes should be interpreted in depression.

## Figures and Tables

**Table 1:** Evidence architecture for ATX/LPA/LPAR involvement in depression and mood-relevant biology through 12 May 2026.

Study/Evidence Category	Cohort/Model/Sample and Region	Main ATX/LPA/LPAR Finding	Interpretive Boundary
**A. Direct human MDD and psychiatric fluid evidence**			
Itagaki et al. [31]; direct human MDD evidence	Serum: 37 MDD patients undergoing ECT and 47 nondepressed controls. CSF: 26 MDD patients and 27 controls. Sample/region: serum and CSF.	Serum ATX and CSF ATX were lower in MDD. Serum ATX increased after ECT and was inversely related to depressive symptom burden.	Supports altered measured ATX levels in clinical MDD; does not identify tissue source or local brain LPC-to-LPA conversion.
Omori et al. [8]; psychiatric fluid evidence including MDD patients	CSF from 26 MDD patients, 27 schizophrenia patients and 27 healthy controls. Sample/region: CSF.	LPA 22:6 was lower in MDD and schizophrenia. Total CSF LPA was less informative. LPA 22:6 was associated with LPC 22:6; CSF ATX activity and PLPP1 did not differ across groups, and LPA 22:6 did not correlate with ATX activity in patient groups.	Supports a species-specific CSF abnormality; does not establish MDD specificity or a simple ATX-driven local conversion mechanism.
Gotoh et al. [33]; direct human MDD negative biomarker evidence	CSF: 52 MDD patients and 49 controls. Plasma: 47 MDD patients and 44 controls. Sample/region: CSF and plasma.	Total LPA showed no association with MDD diagnosis, symptom severity or psychotropic medication.	Argues against total LPA as a general diagnostic or severity biomarker for MDD; does not test molecular species, anatomical ATX pools or receptor output.
Riya et al. [9]; direct human MDD serum evidence	53 MDD patients and 50 matched healthy controls. Sample/region: serum.	Serum total LPA and total LPC did not differ significantly between groups. Serum LPA and LPC correlated positively within the MDD group.	Shows that total serum LPA and LPC are not sufficient for group separation; the within-MDD correlation suggests biochemical coupling, not disease-specific elevation.
Kim et al. [10]; direct human MDD serum lipidomic evidence	Female drug-free subjects. Discovery: pooled serum from 10 current MDD patients, 10 remitted MDD patients and 10 controls. Verification: 25 individuals per group. Sample/region: serum.	LPA 16:1 entered multilipid panels that distinguished current MDD from controls and remitted MDD from controls.	Supports species-resolved lipid signatures in MDD cohorts; does not establish a stand-alone causal axis.
**B. Depression-relevant experimental models**			
Wang et al. [34]; depression-relevant CUMS evidence	CUMS mice; hippocampal AAV-ATX supplementation; HT22-cell assays. Region/sample: hippocampus and cultured neurons.	CUMS reduced hippocampal ATX and LPA. Hippocampal ATX supplementation alleviated depression-like behavior; LPA affected synapse-related proteins and ERK/CREB-linked plasticity readouts.	Supports a depression-relevant regional ATX/LPA mechanism; does not establish clinical MDD evidence or an upstream peripheral LPC route.
Moreno-Fernández et al. [23]; depression-like behavioral model evidence	maLPA1-null mice and wild-type mice after acute LPA1 blockade. Region/sample: behavior and functional brain activity.	Both permanent deletion and acute pharmacological LPA1 inhibition induced depression-like behavior with related brain-activity changes.	Supports behavioral relevance of LPA1 signaling in a depression-like model; does not establish clinical MDD evidence or a completed LPC-ATX-LPA-LPAR chain.
Rosell-Valle et al. [24]; depression-like and anxiety-like ligand/receptor perturbation evidence	Mice receiving chronic central C18:1 LPA or chronic LPA1-3 receptor antagonism. Region/sample: mouse brain and hippocampus.	Chronic C18:1 LPA produced antidepressant-like effects and enhanced adult hippocampal neurogenesis. Chronic LPA1-3 antagonism induced anxiety- and depression-like behaviors and reduced neurogenesis.	Shows ligand exposure, receptor context and neurogenesis dependence in affective-like behavior; does not provide disease-specific MDD evidence.
**C. Broader mood, stress, anxiety, receptor and synaptic physiology evidence**			
Tabbai et al. [21]; stress/anxiety-related receptor model evidence	Wild-type and maLPA1-null mice exposed to acute stress. Region/sample: hippocampus.	Stress and LPA1 deficiency reshaped hippocampal LPA species. Anxiety-related behavior was associated with LPA 16:0, and locomotor measures with LPA 18:0.	Supports stress/anxiety-related receptor-dependent lipid redistribution; does not address MDD diagnosis.
Moreno-Fernandez et al. [22]; chronic-stress receptor-pathway evidence	Mouse chronic-stress model with LPA-LPA1 pathway analysis. Region/sample: ventral hippocampus and behavior.	The LPA-LPA1 receptor pathway modified stress-related behavior and ventral hippocampal excitatory–inhibitory gene profiles.	Supports receptor-linked stress adaptation and circuit balance; does not directly test clinical MDD.
Rosell-Valle et al. [25]; anxiety-like and coping-related receptor phenotype evidence	LPA1-deficient mice with interneuron precursor transplantation. Region/sample: dorsal hippocampus.	LPA1 loss was associated with GABAergic deficits, anxiety-like behavior and coping abnormalities. Interneuron precursor transplantation improved the phenotype.	Connects LPA1 signaling to inhibitory-circuit integrity and coping behavior; remains broader mood/stress evidence.
Moreno-Fernández et al. [26]; stress-system and social-behavior receptor evidence	maLPA1-null mice. Region/sample: social behavior and endocrine stress regulation.	Mice showed social avoidance, a blunted dexamethasone response and abnormal corticosterone rhythmicity.	Extends LPA1 relevance to stress-system regulation; does not provide direct MDD evidence.
Sanchez-Marin et al. [27]; sex-stratified receptor-deficiency evidence	Male and female maLPA1-null mice. Region/sample: plasma lipid signaling, amygdala and medial prefrontal cortex transcriptional readouts.	LPA1 deficiency produced sex-dependent emotional phenotypes and region-dependent neurotransmitter-related transcriptional changes, together with altered plasma LPA/2-AG signaling.	Supports sex and brain region as modifiers of receptor interpretation; not MDD-specific evidence.
Brandt et al. [30]; neuronal physiology evidence	Cultured hippocampal neurons. Region/sample: hippocampal neurons.	LPA modulated excitatory transmission and intracellular calcium responses.	Provides cellular output relevant to synaptic physiology; not disease evidence by itself.
Larrieu et al. [35]; anxiety-vulnerability translational evidence	Humans: 26 controls, 19 high-risk susceptible individuals and 14 high-risk resilient individuals; anxiety-stratified mice. Region/sample: serum, dentate gyrus and adult neural stem/progenitor cells.	Serum LPA 16:0 was higher in high-risk susceptible humans and high-anxiety mice. LPA 16:0 tracked anxiety, reduced adult neural progenitor proliferation and reduced stress resilience through LPA1-dependent mechanisms.	Supports endogenous LPA 16:0 involvement in anxiety vulnerability and stress resilience; not direct MDD-specific evidence.
Tuscher et al. [29]; local lipid-signaling and psychiatric-phenotype evidence	Human PRG-1 R345T carriers and Prg-1 mutant mice. Region/sample: cortical synaptic physiology, fear/memory tasks and mouse behavior.	PRG-1 dysfunction produced intermediate psychiatric phenotypes in humans and increased anxiety, depressive features and low stress resilience in mice. ATX inhibition normalized selected mouse phenotypes.	Supports local ATX-linked lipid-signal excess and synaptic dysfunction; not a bulk-fluid biomarker or MDD-specific pathway.

**Table 2:** Direct-evidence requirements for pathway-level validation of the LPC-ATX-LPA-LPAR sequence in depression-relevant biology.

Layer	Minimum Measurement	Model-Supporting Pattern	Model-Weakening Pattern
Fluid lipid layer	Plasma, serum or CSF PC/LPC/LPA species, not only total LPC or total LPA.	Defined species differ across compartments, such as CSF LPA 22:6 changing while total LPA is unchanged.	Only total lipid values are measured, or species changes do not replicate.
CNS substrate-enzyme encounter layer	Defined LPC species, anatomical ATX localization or activity, and where feasible labeled substrate tracing or spatial lipidomics in interface and parenchymal compartments.	A predicted LPC species and catalytically active ATX converge in the same CNS compartment, with local LPA production linked to the downstream model.	LPC species and active ATX are not spatially linked, or interface pools cannot be connected to parenchymal output.
Local production-inactivation layer	ATX abundance, ATX activity, local LPA species and, where relevant, PLPP1/LPA-dephosphorylating activity in brain region or interface tissue.	Local LPA species relate to a defined production-inactivation state more strongly than to total fluid lipid levels.	Local ATX activity, LPA species and LPA inactivation readouts do not relate to receptor output or behavior.
Receptor output layer	LPAR subtype expression, calcium signaling, PLC/PKC, E/I markers, synaptic readouts.	Specific LPAR subtype output changes in the predicted brain region or cell type.	Receptor signaling changes are absent or nonspecific.
Behavior or circuit layer	Depressive-like behavior, stress adaptation, electrophysiology or imaging readouts.	Pathway perturbation modifies both molecular output and behavior or circuit readout.	Perturbation changes molecular markers but not behavior or circuit function.

**Table 3:** Testable and non-testable formulations for evaluating the LPC-ATX-LPA-LPAR axis in MDD.

Research Focus	Testable Formulation	Overly Broad or Unfalsifiable Formulation to Avoid
LPC substrate availability and ATX-mediated LPA generation	In the same MDD cohort or depression-relevant model, a defined LPC species should be examined together with ATX abundance or activity and a corresponding LPA species in the same sample type or predefined brain region. If LPC changes are not accompanied by ATX activity or corresponding LPA species changes, this would weaken the interpretation that altered LPC availability supports ATX-mediated LPA generation in that setting.	Lipid changes may contribute to LPA signaling in depression.
ATX abundance versus ATX enzymatic activity	ATX abundance and ATX enzymatic activity should be measured separately. If ATX abundance changes without corresponding changes in ATX activity or LPA species in the same biological setting, this would not support ATX-mediated LPA production as the main explanation for that finding.	ATX is involved in depression.
Local LPA production, inactivation and LPAR subtype involvement	A pathway-level interpretation would require evidence that local LPA production or inactivation and a specific LPAR subtype are altered in the same depression-relevant model, brain region or cell population. If LPA species changes are not accompanied by LPAR subtype changes or receptor-related functional outcomes, the evidence would support a lipid alteration but not a complete LPC-ATX-LPA-LPAR pathway.	LPA receptors may participate in mood regulation.
Relationship to MDD phenotypes	In human studies, LPC species, ATX activity, LPA species or LPAR-related readouts should be analyzed in relation to MDD diagnosis, symptom dimensions, treatment status or longitudinal change, with attention to sample type, medication status, sex, age and other relevant covariates, including reproductive stage when relevant. If these variables show no association with predefined MDD-related clinical measures, the proposed MDD relevance in that cohort would be weakened.	The LPC-ATX-LPA-LPAR axis may be important in MDD.
Temporal order and causal testing	Longitudinal clinical studies, time-course experiments or targeted manipulation of ATX, LPA metabolism or LPAR subtypes are needed to test whether LPC changes precede ATX/LPA changes and whether these changes precede receptor-related neural or behavioral outcomes. If temporal or intervention studies do not support this order, the pathway should not be described as a sequential mechanism in that setting.	LPC changes lead to LPAR-mediated depressive symptoms.

## Data Availability

No new data were created or analyzed in this review. This article uses purposive synthesis of published literature, and data sharing is not applicable.
